# Sooty tern (*Onychoprion fuscatus*) survival, oil spills, shrimp fisheries, and hurricanes

**DOI:** 10.7717/peerj.3287

**Published:** 2017-05-10

**Authors:** Ryan M. Huang, Oron L. Bass Jr, Stuart L. Pimm

**Affiliations:** 1 Nicholas School of the Environment, Duke University, Durham, NC, United States of America; 2 South Florida Natural Resources Center, Everglades National Park, Homestead, FL, United States of America

**Keywords:** Climate change, Fisheries, Migration, Oil spill, Seabirds, Telemetry, Survivorship

## Abstract

Migratory seabirds face threats from climate change and a variety of anthropogenic disturbances. Although most seabird research has focused on the ecology of individuals at the colony, technological advances now allow researchers to track seabird movements at sea and during migration. We combined telemetry data on *Onychoprion fuscatus* (sooty terns) with a long-term capture-mark-recapture dataset from the Dry Tortugas National Park to map the movements at sea for this species, calculate estimates of mortality, and investigate the impact of hurricanes on a migratory seabird. Included in the latter analysis is information on the locations of recovered bands from deceased individuals wrecked by tropical storms. We present the first known map of sooty tern migration in the Atlantic Ocean. Our results indicate that the birds had minor overlaps with areas affected by the major 2010 oil spill and a major shrimp fishery. Indices of hurricane strength and occurrence are positively correlated with annual mortality and indices of numbers of wrecked birds. As climate change may lead to an increase in severity and frequency of major hurricanes, this may pose a long-term problem for this colony.

## Introduction

Given a variety of threats to ocean health, ranging from climate change to pollution and overfishing (Campagna et al., 2011; Sissenwine, Mace & Lassen, 2014; Boonstra et al., 2015), assessing ocean conditions is vital (Knap et al., 2002; Samhouri et al., 2012). Monitoring colonial seabirds has provided a method of ocean threat assessment (Le Corre & Jaquemet, 2005). Scientists have been observing nesting seabird populations for decades (Thienemann, 1902; Lockley, 1934). Seabirds provide researchers large, consistent datasets with relative ease and permit the marking of many individuals. Unfortunately, while what happens in the nesting colonies may be understood in exceptional detail, what happens at sea has generally only been known indirectly from year-to-year changes in the nesting birds or from the locations of banded individuals found wrecked and deceased away from the colony (Piatt & Van Pelt, 1997; Schreiber, 2002). Even where the birds feed—both when at the colony and during the rest of the year—has often been poorly known.

Only recently has a large variety of technological options allowed scientists to track seabirds away from the colony (Jouventin & Weimerskirch, 1990; Burger & Shaffer, 2008; Le Corre et al., 2012; Dean et al., 2015). From geolocators that record day length and sunrise to platform terminal transmitters (PTTs) that transmit location data to satellite arrays, more tools allow increasingly more accurate observations on more species. These technologies allow researchers to connect knowledge on annual survival to physical locations where nesting birds feed and disperse at other times of the year.

The US National Park Service (NPS) established an Inventory and Monitoring Program to provide park managers and others more scientifically credible information on the status and trends of natural resources (Patterson et al., 2008). Their goals include providing early warning of abnormal environmental conditions and determining whether human activity is responsible for such changes. In this study, we respond to the NPS’ needs by monitoring a large breeding colony of roughly 80,000 *Onychoprion fuscatus* (sooty terns) in the Dry Tortugas National Park, located in the Florida Keys. For the Dry Tortugas National Park, the specific concerns are fisheries—a large pink shrimp industry that has operated in the area for the last 60 years (NMFS, 2016)—and the world’s worst oil spill, Deepwater Horizon, the source of which occurred approximately 800 km to the northwest in 2010 (MacDonald et al., 2015). While the effects of oil spills on seabird mortality are well-documented (Burger, 1993; Votier et al., 2005; Votier et al., 2008; Moreno et al., 2013), the impact of the shrimping industry is less straightforward. Commercial fisheries may have complex relationships with seabirds, from direct mortality from fishing equipment to enhanced food availability from unwanted discards (Tasker et al., 2000; Furness, 2003). Research specifically on the impacts of commercial shrimping using trawl gear suggest such activity acts as a strong local attractor to seabirds via increased food availability (Garthe, Camphuysen & Furness, 1996), but can alter interspecies competition by preferentially supporting gulls and pelicans as opposed to smaller species such as terns (Garthe & Scherp, 2003; Wickliffe & Jodice, 2010).

In addition to potential anthropogenic impacts, natural disturbances in the form of tropical storms are likely to influence seabird mortality. Given that hurricanes are catastrophic disturbances that can cause extensive damage to both terrestrial and marine habitats (Lodge & McDowell, 1991; Nystrom, Folke & Moberg, 2000; Vandermeer et al., 2000), one might reasonably expect such storms to impact seabirds, both at the colony and wrecking individuals during migration at sea (Schreiber, 2002). Moreover, current estimates of climate change predict an increase in severity and frequency of category 4 and 5 hurricanes (Bender et al., 2010) thus potentially magnifying such effects.

Here, we use a combination of current satellite tracking techniques and historical banding data to achieve the following objectives: (1) estimate annual mortality, (2) map annual movements of sooty terns from the Dry Tortugas, (3) determine the extent of overlap between sooty tern distribution during the breeding season and commercial shrimping and the 2010 Deepwater Horizon oil spill, and (4) better understand the consequences of episodic major hurricanes on mortality and incidences of wrecked individuals. These objectives stem from a lack of knowledge on sooty tern populations and at-sea movements. Unfortunately, as with many catastrophes, data collection does not happen until after the incident occurs, so we must compare the past Deepwater Horizon incident with present telemetry data to determine the potential overlap such a future spill might incur. Although many studies have looked at the annual survival and dispersal of other terns (Coulson & Horobin, 1976; Møller, 1983; Renken & Smith, 1995; Spendelow et al., 1995; Nisbet & Cam, 2002; Szostek & Becker, 2012; Breton et al., 2014; Szostek & Becker, 2015), there are few, if any, capture-mark-recapture (CMR) studies that include telemetry data and are specific to sooty terns. There are no previous such studies for this colony. In addition, while studies have illustrated the impact of individual cyclones on the mortality, reproduction, and demography of birds at their colonies (Wunderle & Wiley, 1996; Genovart et al., 2013; Oro, 2014), few studies have estimated the direct impact of hurricanes during the birds’ movements away from the colony (Nicoll et al., 2016).

## Methods

### Study site, species, & historical banding

Sooty terns are an abundant seabird found in large colonies in the Atlantic and Pacific oceans (Bird Life International, 2017). While there have been many studies on sooty terns on various islands in the Indian Ocean (Feare, 1976; Feare & Doherty Jr, 2004; Young et al., 2010) and a well-studied colony on Ascension Island (Ashmole, 1963), the Dry Tortugas National Park is the longest continuously studied sooty tern nesting ground in the Atlantic Ocean (1959 to the present). Located at 24.63°N and 82.87°W, the Dry Tortugas consist of seven keys, of which sooty terns only breed on one, Bush Key. Roughly 80,000 individual sooty terns to breed each year. For the majority of sooty terns at the Dry Tortugas, the breeding season lasts from February to May, but some may arrive as early as December and some leave as late as August. Our current knowledge of the sooty terns at the Dry Tortugas comes from a combination of a long term banding effort and studies recording the behavior, energetics, growth, and chick mortality (Watson, 1908; Sprunt Jr, 1948; Robertson Jr, 1964; Dinsmore, 1972). As is typical with many seabird studies, most of the past work has been limited to observations at the colony. Until recently, we did not know even where the birds fed when nesting.

Marking of sooty terns at the Dry Tortugas started in 1937. Since 1959, researchers have marked just under half a million birds. Each year there were two main banding efforts, one that took place shortly after adults arrived and laid their eggs and a second that occurred about six to seven weeks before most of the chicks fledged. Adults were caught using a combination of hand nets and mist nets; for the juveniles, the banders would herd the flightless chicks into corrals made of chicken wire where teams would then band as many as possible. This method of mass banding adults lasted until 1974.

### Telemetry and movement analysis

Before the use of modern telemetry technology, our insight into bird movement came predominantly from recapturing banded individuals or recovering bands from deceased ones. As a first step to mapping migration, we first gathered all available data on recovery locations of bands put on sooty terns at the Dry Tortugas from the Bird Banding Laboratory (BBL) database (https://www.pwrc.usgs.gov/bbl/). We then mapped the recoveries of dead of sooty terns away from the breeding colony. To show the temporal and spatial distribution, we aggregated the recovery location information into regional clusters and subdivided them into three month periods (January to March, April to June, July to September, and October to December).

To gain a better understanding of where sooty terns live and not simply where they die, we used two types of telemetry devices. In both cases, we caught individuals using hand nets on Bush Key in the Dry Tortugas. In March 2011, we attached 25 1.2 g BAS-MK10 GLS light level geolocators to adult sooty terns. Average adult sooty terns are about 200 g, so these geolocators are about 0.6% of their body weight. These geolocators record day length and relative time of sunrise. With these two pieces of information, we used the BASTrak software provided by the British Antarctic Survey to estimate the latitude and longitude for each day. Of the 25, we were only able to reclaim two geolocators the following year. In addition, the logger’s inability to determine latitude accurately during the spring and fall equinoxes (when everywhere experiences 12 h of sunlight) forced us to remove data three weeks before and after these dates.

**Figure 1:**
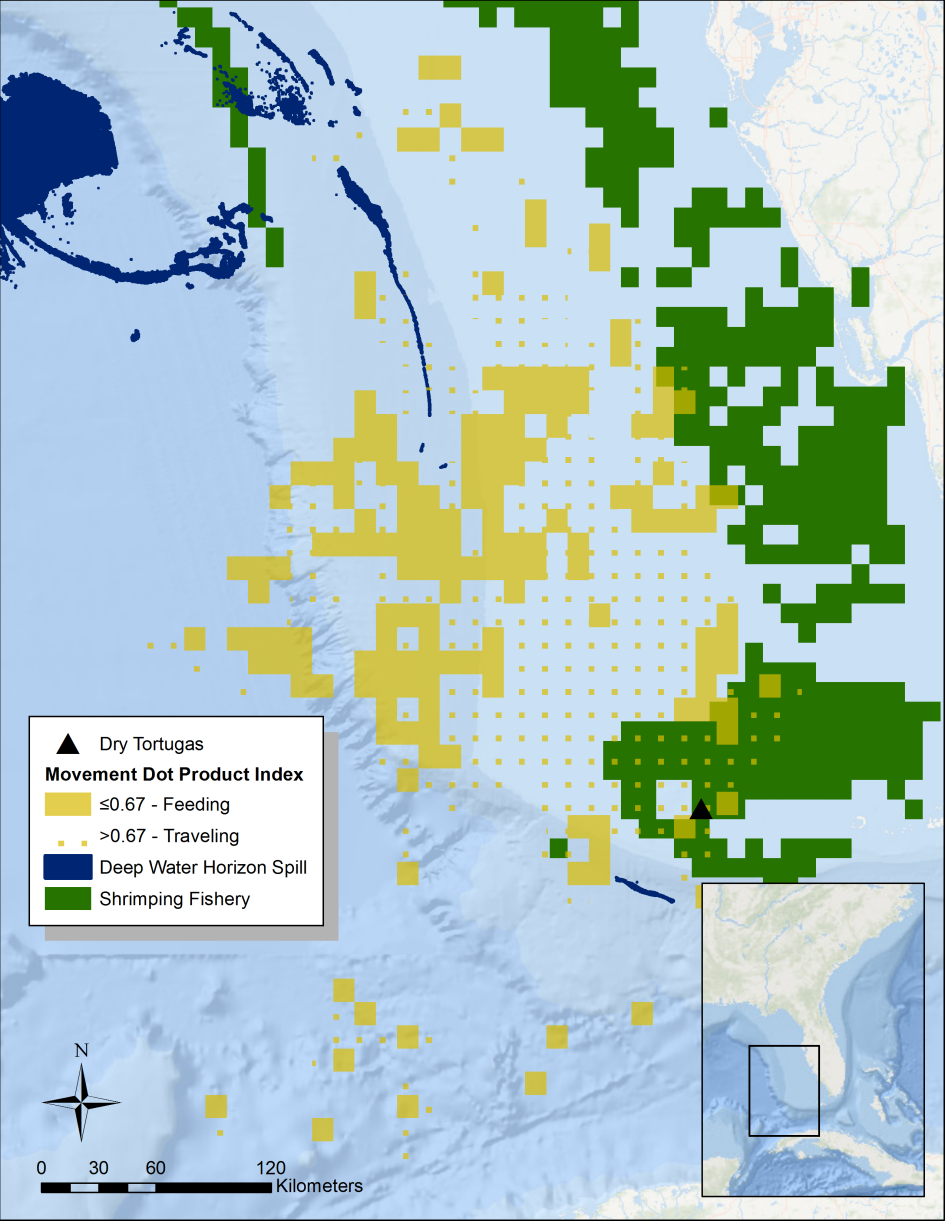
Summary of tern movement during the breeding season in 2014. We calculated DPI using the ArcMET extension for ArcGIS across 0.1 decimal degree cells for three different individuals with satellite transmitters. The DPI is a number between 0 and 1 indicating how aligned tracks are within a given grid cell (Wall et al., 2013). The closer the value is to 0 (solid yellow area), the more dissimilar the tracks are, indicating foraging grounds. We have overlaid these data with data of shrimping locations (solid green; Gallaway et al., 2003) in addition to the surface oil from the Deepwater Horizon spill (dark blue; NOAA, 2010). Basemap is provided by Esri©.

In February 2014, we attached newly available 5 g solar PTT’s from Microwave Telemetry Inc. to three adults using leg loop harnesses. These transmitters weighed only 2.5% of an adult sooty tern’s body weight. We set the transmitters to transmit hourly data with an off cycle of either 48 h or the length of time it took to fully charge the battery via the solar panel, whichever was shorter. This was done to maximize the amount of data we could retrieve. One PTT failed after two weeks, another after six months, and the third lasted until September 2015 (allowing for partial data collection on a second year of migration). We retrieved location data from the ARGOS satellite system and archived the dataset at http://www.movebank.org. Data from the PTT’s fall into location classes based on the estimated error. We discarded all locations with an error greater than 1,500 m (the maximum error ARGOS could tolerate and still give a reliable location). We then calculated the speed and heading of tracks between points less than a day apart. Both the banding and the satellite tagging were performed under federal bird banding permit 21780.

To identify potential foraging areas, we used a dot product index (DPI) analysis from the ArcMET extension for ArcGIS v2.3 to summarize the average speed and heading of all the tracks of the three individuals with satellite transmitters (Fig. 1). We summarized tracks across 0.1 decimal degree grid cells during the entire 2014 and 2015 breeding seasons (February to May). The DPI is a number between 0 and 1 indicating how aligned tracks are within a given grid cell (Wall et al., 2013). The closer the value is to 1 (dotted yellow area in Fig. 1), the more directionally consistent the tracks in the cell are, very likely indicating the individuals are moving to and from the breeding grounds. Conversely, cells with tracks of divergent directions have a DPI value closer to 0 (solid yellow area in Fig. 1), and most likely represent searching for food—i.e., the bird is moving back and forth searching for prey. Given that this analysis is a summary across two entire breeding seasons, we assume that daily environmental conditions such as wind speed become unimportant. For the DPI analysis, we considered any tracks that had points that were more than 24 h apart as separate and used the Jenks natural break optimization to classify DPI values of individual grid cells into a predominantly traveling or foraging location. We then overlaid these data with those collected on shrimp boat movements and trawling locations from 2003 to 2014 (solid green area in Fig. 1; Gallaway et al., 2003). The peak season for commercial shrimping in the Florida Keys lasts from January to June, thus temporally overlapping with the sooty tern breeding season. Additionally, we looked at the overlap with the location of surface oil from the Deepwater Horizon oil spill (NOAA, 2010). Since we are only interested in determining which areas were impacted by the spill, we ignored the day-to-day variation and identified all areas that experienced at least one day of surface oil. To calculate overlap between areas used by track individuals and areas impacted by humans, we simply divided the affected area by the area used (the total area from all 0.1 decimal degree cells that contain tracks).

### CMR mortality analysis

We calculated estimates of mortality using a single-state Cormack–Jolly–Seber (CJS) model implemented in Program MARK (Lebreton et al., 1992; White & Burnham, 1999) where both the survival (*φ*) and recapture probability (*p*) were time-dependent. Since this is an open population model, this method only calculates apparent survival and some individuals in the population might have emigrated rather than died. For simplicity, we also removed approximately 200 individuals who were recovered deceased, so the model was not a mix of known and unknown fates. This left us with capture histories for 103,076 individuals. We tested the simple model of only a year effect against two other models, one that assumed an age-dependent effect and another that assumed a cohort effect (using an all different parameter matrix for *φ* in MARK; Cooch & White, 2002). While the age-dependent model might pick up differences in survival between younger and older individuals, we created the cohort model to determine if newly banded birds might have increased or decreased survivorship after a strong hurricane season. We then re-ran the models, and linearly constrained them with measures of annual cumulative wind speed of hurricanes (calculated by summing the maximum sustained wind speed over each six hour period). Since the cohort model uses an all different parameter matrix, we were unable to run a linearly constrained model in MARK. Using the U-CARE program (Choquet et al., 2009), we assessed the goodness-of-fit and used the resulting ĉ to adjust the Akaike’s Information Criteria (AIC) for over-dispersion due to binomial noise. We ranked the final models based on the resulting QAICc (quasi-likelihood AIC; Burnham & Anderson, 2002). In addition to using MARK to generate estimates of mortality, we also developed our own CMR model specific to our population to identify why certain years differed and provide a transparent understanding and visualization of the basic mechanisms at work within the MARK software (see Supplemental Information for results and details).

One assumption of CMR studies is that captured individuals are a random sample of the population and thus are representative of the population as a whole. However, our data suggest this might not be entirely true for our study (see Supplemental Information). Some individuals appear more likely to be caught multiple years in a row, thus biasing the detection rate. While we are able to account for this in our supplemental model—and it does not alter our conclusions—it is unclear how this may affect estimates provided by MARK.

As we show below, juveniles do not return to the breeding colony for at least four years after fledging, so we do not calculate their survival. Juveniles re-captured when they return to the colony as adults are treated as adults banded for the first time. Due to limited sample size and the change in sampling methodology, we have limited our analysis of the banding data to the period from 1960 to 1974. Inspection of the band numbers for 1959 suggests that many band numbers were absent from the data submitted, so we excluded this year.

### Estimation of mortality from wrecked individuals

In conjunction with our estimates of mortality from CMR data, we calculated an alternative, independent proxy of mortality using the data from recovered, deceased individuals taken from records available from 1960 to 1980. These records come from the BBL database of bands originally put on sooty terns at the Dry Tortugas and later recovered. Such instances of mortality can be caused by starvation or exhaustion after a storm event and are known as “wrecks” (Piatt & Van Pelt, 1997; Schreiber, 2002). For this study, we have defined a “wrecked” individual as a recovered, deceased sooty tern found in a location that we considered unlikely to have been a part of their normal migration route (identified as the route taken by the individuals tagged with PTT’s and geolocators). For each year, we calculated the number of individuals known to be alive given that they were captured again in a later year and then estimated mortality using a simple proportion of the number of recovered individuals out of the number known alive in that year. Since the detection rate of wrecked individuals is unknown, spans a large geographic area, and the known alive population is likely an underestimation because of a low recapture rate, this method of estimating mortality is a proxy. Lastly, to compare these estimates with those derived from the MARK model we ran a correlation analysis using the inverse of the standard errors of the MARK estimates as weights.

### Hurricane impact

To attempt to identify potential storm-induced mortality, we identified notable cyclones in two different ways. The first was that for any year where wrecked birds were recovered, we identified hurricanes whose paths both overlapped with the wrecked locations and took place before the recovery. Wind swath data are not readily available before 2008. Thus, we used an estimate of 80 km from the storm center to the eye wall for hurricane-force winds and an estimate of 320 km from the storm center for gale force winds (Tannehill, 1934). Since hurricane size is independent of intensity, the wind radii estimates are the same along the entire storm’s track (Tannehill, 1934). We created maps of these storms and wrecked locations to create a visual representation of the correlation in addition to calculating the distance between wrecks and a storm’s center and the amount of time between a storm’s formation and the recovery of such wrecks.

For the second method of estimating the impact of storms, we selected all hurricanes that were category 3 or above, between the years 1960 and 1980, that at least partially overlapped with the region of interest (as determined by the full spatial extent of locations from both the PTT’s and geolocators). Using this selection of storms, we ran a simple linear regression between the number of wrecked individuals in a given two-week period and the number of storms in the same period as well as a linear regression with the cumulative wind speed of such storms. For years for which we had an estimate of mortality from the constrained year only MARK model (1960–1974), we also ran a weighted linear regression between the average estimates of mortality and the cumulative wind speed of our selection of storms across the year using the inverse of the variances from MARK as the weights. We did not use the accumulated cyclone energy (ACE; Bell et al., 2000) indices in our analysis since we were only interested in storms in one region of the Atlantic Ocean and simple cumulative wind speed allows us to break storms down by weeks and months rather than just years.

## Results

### Movements during the breeding season

Altogether, during this study we successfully tracked five individual sooty terns, two during the 2011 breeding season using geolocators, three during the 2014 breeding season using PTT’s. One PTT-tracked tern only contributed two weeks of data but another individual with a PTT continued into the early part of its migration in 2015. This provided us data from two complete breeding seasons, but since the individual had a similar pattern of movement in both years, we have summarized all the data here. From January through May, the tagged individuals spent most of their time to the northwest of the island, reaching areas as far away as 415 km and traversing over 58,222 km ^2^ of open water along the continental shelf (Fig. 1). The Jenks natural breaks analysis split the DPI values at 0.67, which resulted in classifying 39.4% (22,955 km ^2^) of the total area used by the individuals as feeding grounds. Areas closest to the island are mostly directionally consistent, which suggests that the individuals are traveling back and forth (Fig. 1). The cells 250 km to the northwest however, are directionally variable, suggesting this where the birds feed. Overall, only 9.2% of the total area the study individuals used overlapped industrial shrimping (Gallaway et al., 2003), and 20.1% of this overlap was foraging grounds, representing only 4.7% of the total foraging area.

Although the Deepwater Horizon well was roughly 800 km away from the Dry Tortugas, trace amount of surface oil (roughly 16 km ^2^) did overlap with the area that study individuals used for traveling but not for foraging, as estimated by the DPI analysis (Fig. 1).

### Movements during migration

**Figure 2:**
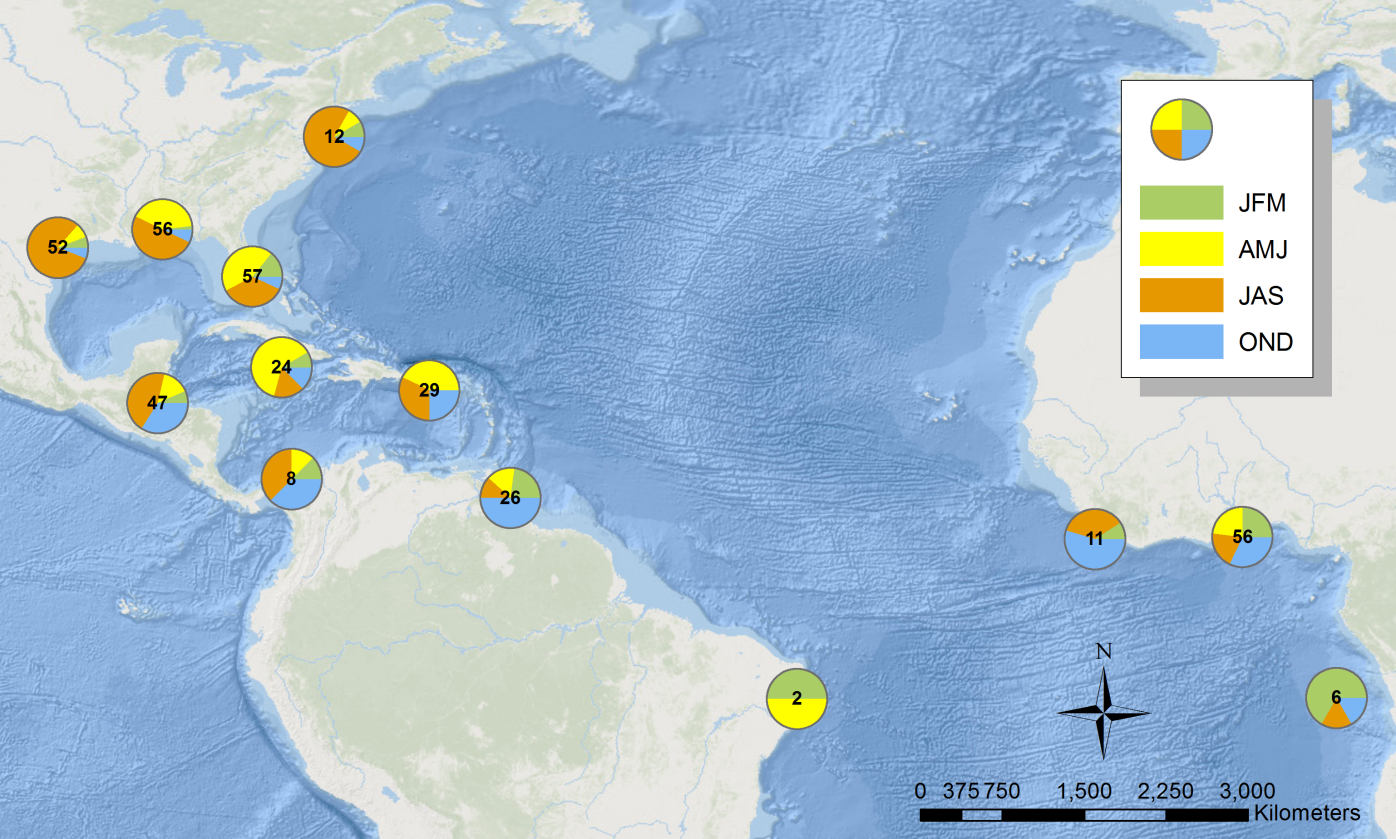
Map illustrating the location of sooty terns banded at the Dry Tortugas recovered in the Atlantic Ocean. Each pie shows the proportion of individuals in the area found in each season. The number in the center is the total number of records. Basemap is provided by Esri©.

Before the use of geolocators or PTT’s, we could only approximate the sooty tern migration across the Atlantic Ocean using data from 386 recovered individuals from the BBL data that stretch back to 1937 (Fig. 2). Individuals found in the Gulf of Mexico are predominantly between April and September while those recovered in the Caribbean Sea tend to be between June and December. Lastly, terns retrieved from the coast of Africa are usually between October and March. While 51% of all band recoveries were from juveniles, a disproportionate 89% of the recoveries in Africa were from juveniles. This supports previous work that suggests these individuals found in Africa are mostly juveniles that have yet to return to the colony to breed (Robertson Jr, 1969). A full breakdown of when and where terns were recovered can be found in Table S1.

**Figure 3:**
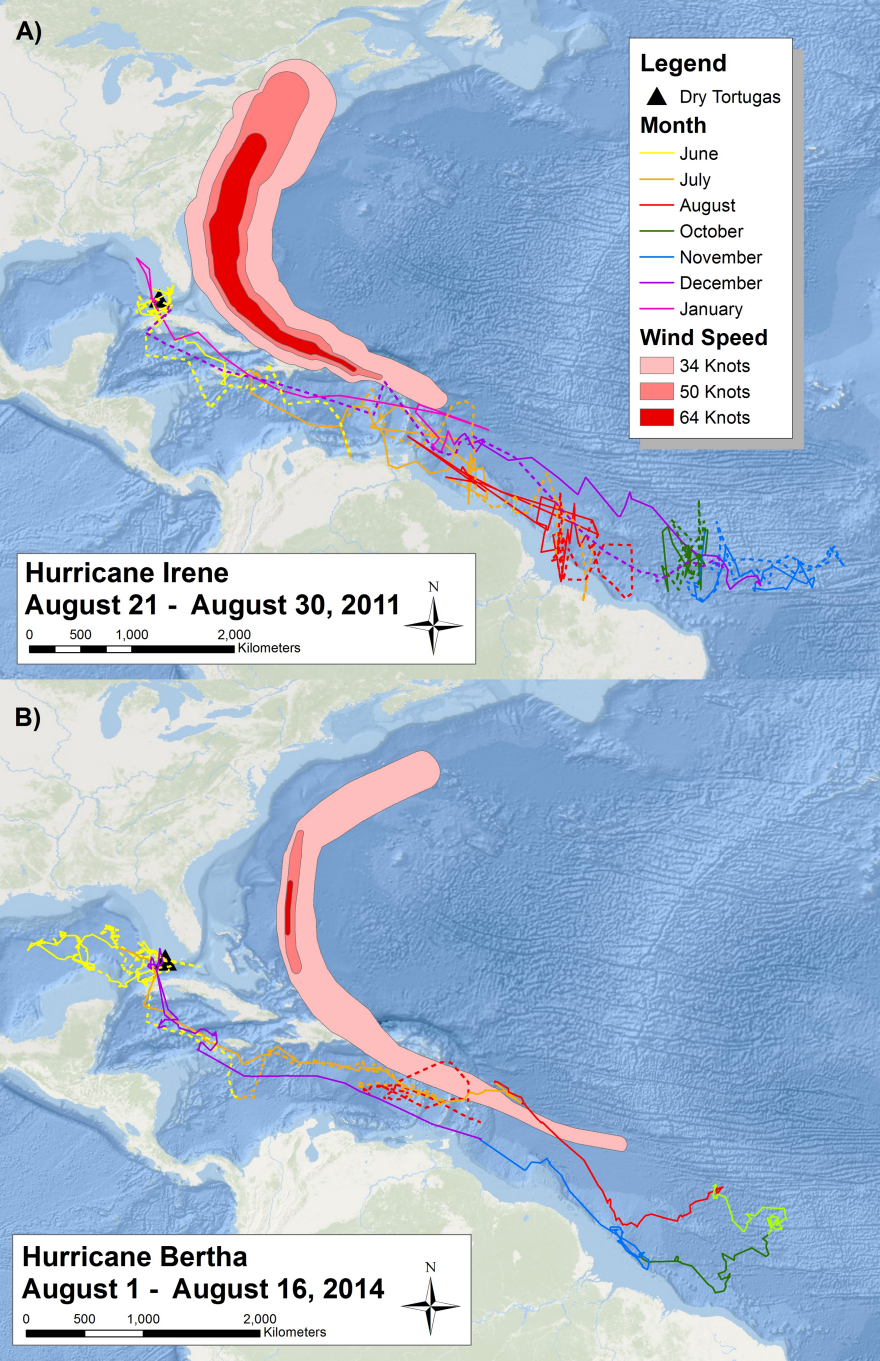
Migratory route of tagged sooty terns in 2011 and 2014. Two maps reporting the daily movements of sooty terns tagged with (A) geolocators and (B) PTT’s throughout their migration. Each map has the tracks of two different individuals (one with a solid line and one with a dashed line) color coded by month. We have not included September data from 2011 due to the impact of the vernal equinox on the accuracy of the geolocators. The proximity of the area affected by Hurricanes Irene and Bertha with the sooty tern movement tracks of the year illustrates the potential spatial and temporal overlap between hurricanes and the tern migration. Basemaps provided by Esri©.

With the use of geolocators and PTT’s, we can understand the migratory route of sooty terns in detail. The two individuals with geolocators provided data on a complete migration after leaving the island in 2011 and returning in 2012 (Fig. 3A), but we collected more detailed data from the two individuals with PTT’s who started their migration in 2014 (Fig. 3B). Due to transmitter failure, data from one individual with a PTT ended in August, providing an incomplete migration map. All birds spent most of June traveling around the Gulf of Mexico before traveling through the Yucatán Channel at the end of June or beginning of July. The terns then spent July passing through the Caribbean Sea before reaching the Lesser Antilles around the start of August. Figure 3 also illustrates the wind swaths of Hurricane Irene and Hurricane Bertha during August. The tracks of both birds in 2014 overlap geographically and temporally with Hurricane Bertha while those in 2011 just narrowly avoided Hurricane Irene to the north, indicating the potential danger sooty terns face on their migration route. We still received movement data from the second individual with a PTT for several days after Hurricane Bertha passed; thus, we do not think that the hurricane was directly responsible for transmission termination. After passing the Lesser Antilles, tagged individuals spent the remainder of August, September, October, and November in the deep waters of the Atlantic Ocean off the northeast coast of Brazil. There are no September data for the individuals with geolocators due to poor data quality from the vernal equinox. While the majority of individuals tagged returned to the Dry Tortugas at the end of December, one individual with a geolocator did not return until mid-January. The timing of these movements is broadly similar to the timing of recoveries seen in Fig. 2 as evidenced by the majority of individuals recovered in the Florida Keys found during the spring months, individuals found in the Gulf of Mexico during the summer, and lastly individuals recovered throughout the Caribbean and South American coast found during fall and winter. Throughout this entire route, the two individuals with PTT’s spent very little time over coastal shelves, preferring deeper waters.

### Estimates of mortality

After running the general time-dependent, year only model, we ran the goodness of fit and adjusted all models based on a ĉ of 2.855. Of the five models run in MARK, both the unconstrained and constrained age-dependent models performed the most poorly (Table 1), suggesting that once an adult, survivorship does not change with age. This corroborates the results of Nisbet & Cam (2002) who found that number of years into adulthood in common terns does not affect survivorship. The simple time-dependent model that was linearly constrained by cumulative wind speed performed the best (Table 1) but was very similar to the unconstrained time-dependent model. Given that the constrained year only model performed the best, we have chosen to report the estimates from this model and used these estimates for all subsequent analyses.

**Table 1:** Model evaluation of three different CMR models generated in MARK (White & Burnham, 1999). Models are ranked by QAICc (quasi-likelihood Akaike’s Information Criteria).

Model	QAICc	ΔQAICc	QAICc weight	QDeviance	# of parameters
Year + Wind	89501.23	0	0.99995	2165.63	38
Year	89521.05	19.82	0.00005	2187.46	37
Cohort	89641.27	140.04	0	1954.98	213
Age + Wind	89730.79	229.56	0	2395.19	38
Age	89730.85	229.62	0	2395.26	38

**Figure 4:**
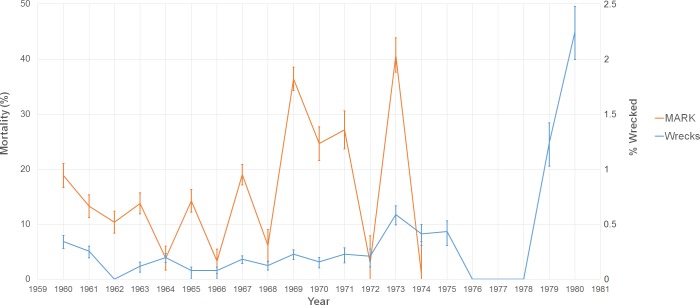
Graph of two estimates of tern mortality. The orange line shows the estimate of mortality from the time-dependent survivorship model generated using MARK. The blue line shows an independent estimate from wrecked individuals. We calculated % wrecked as the proportion of wrecked individuals out of the total number of banded individuals known alive in a given year.

Over the period 1960–1974, the study banded 103,076 adults with 24,530 of them recaptured at least once (Table 2). The average annual apparent mortality and recapture probability during this time were 15.68% (*n* = 15, sd = 11.82%) and 6.48% (*n* = 15, sd = 1.97%), respectively. The maximum and minimum mortality observed were in consecutive years: 41.72% in 1973 and 0.84% in 1974 (Fig. 4). 1967 had the highest number of individuals banded, but the highest recapture probability occurred in 1969 (10.96%). Across the entire study, 295,996 juveniles were caught, but only 21,643 were recaptured as adults (7.31%), and 2,334 of these recaptures (10.78%) were caught four years after banding. Given the similarity between this recapture rate of juveniles and the average detection rate for adults, in this instance we assume that the recapture rate matches the detection rate and that the overwhelming majority of juveniles return to the island in their fourth year. These results match those of juvenile sooty terns studied on Johnston Atoll (Harrington, 1974).

**Table 2:** Banding data of adult terns from 1960 to 1974. Recapture probabilities and mortality estimates were calculated from a time-dependent CMR model in MARK (White & Burnham, 1999).

Year	Captured	Recaptured	Recapture probability (*p*)	Mortality (1 − *φ*)
1960	7,249	0	0.0463	0.1883
1961	7,088	273	0.0498	0.1326
1962	8,226	561	0.0597	0.1034
1963	7,813	1,041	0.0645	0.1378
1964	7,686	1,406	0.0560	0.0381
1965	7,383	1,586	0.0585	0.1422
1966	6,835	1,794	0.0895	0.0324
1967	11,710	3,247	0.0927	0.1899
1968	9,950	3,600	0.0467	0.0614
1969	5,107	2,139	0.1096	0.3639
1970	6,228	3,547	0.0619	0.2462
1971	1,689	1,800	0.0712	0.2713
1972	2,281	1,598	0.0460	0.0294
1973	2,158	1,103	0.0815	0.4072
1974	1,626	1,263	0.0387	0.0084

When we look at our proxy of mortality from the data on wrecked individuals, there is a significant positive correlation with estimates from our MARK model (*r*^2^ = 0.286, *p* = 0.040). Our two estimates show similar patterns of mortality with peaks occurring in 1960, 1967, 1969, 1971, and 1973. Exceptions to the trend occurred in 1964 when there was a peak in the fractions of wrecked birds, but the mortality estimate was low. The opposite occurred in 1965 (where there was a peak in mortality, but the number of wrecked birds was relatively low).

### Hurricane correlations

**Figure 5:**
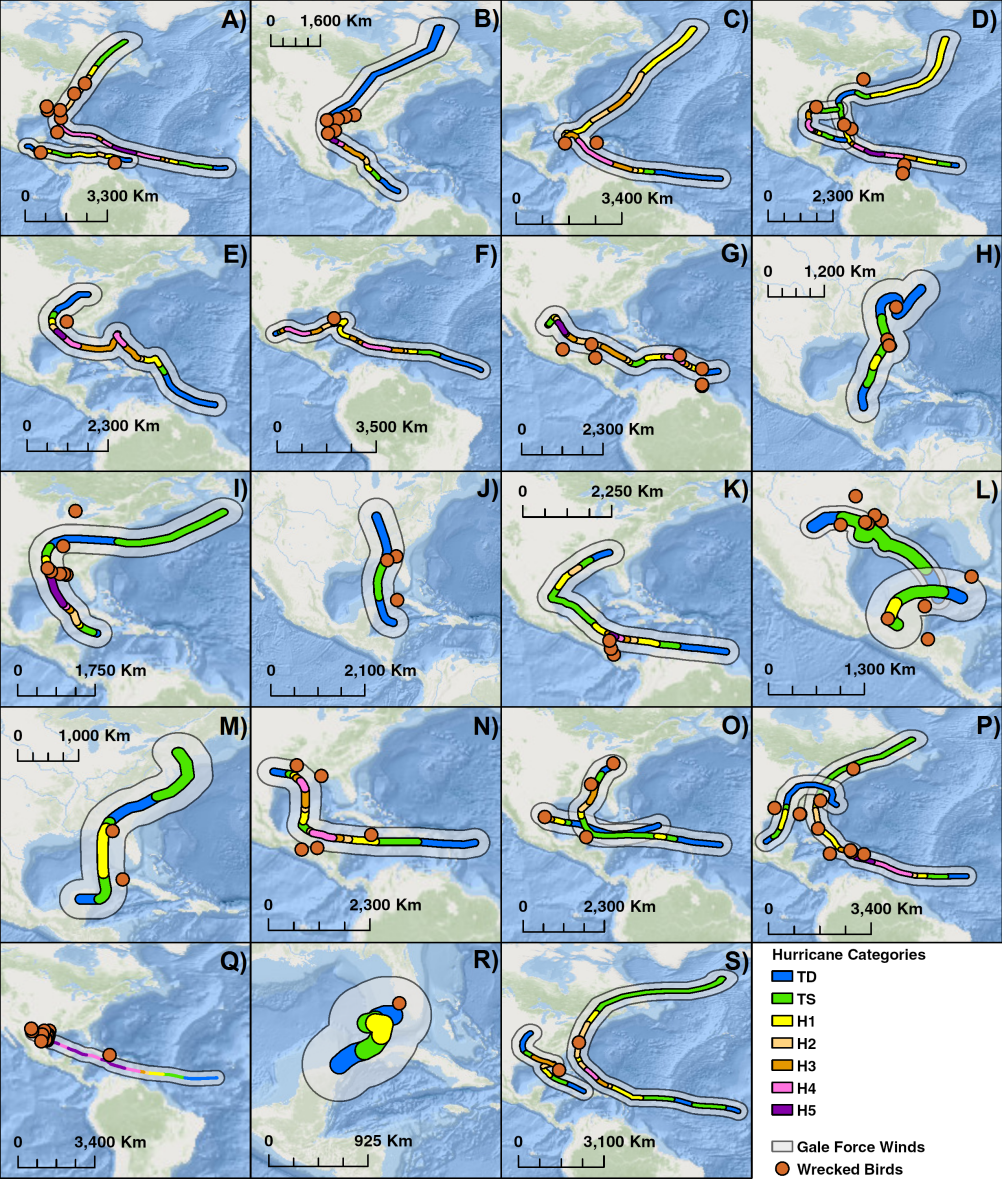
Compilation of maps of 19 years with notable hurricanes and recovered wrecked birds. Hurricanes are color coded by intensity and an estimate of wind swath. (A) 1960: Hurricanes Abby & Donna, (B) 1961: Hurricane Carla, (C) 1963 Hurricane Flora, (D) 1964 Hurricanes Cleo & Hilda, (E) 1965: Hurricane Betsy, (F) 1966: Hurricane Inez, (G) 1967: Hurricane Beulah, (H) 1968: Hurricane Abby, (I) 1969: Hurricane Camille, (J) 1970: Tropical Storm Becky, (K) 1971: Hurricane Edith, (L) 1973: Hurricane Brenda & Tropical Storm Delia, (M) 1972: Hurricane Agnes, (N) 1974: Hurricane Carmen, (O) 1975: Hurricanes Caroline & Eloise, (P) 1979: Hurricanes Bob & David, (Q) 1980: Hurricane Allen, (R) 1982: Hurricane Alberto, (S) 1985: Hurricanes Elena & Gloria. Basemaps are provided by Esri©.

Over the course of 25 years, 25 different storms were associated with 106 wrecked terns (Table 3). We compiled maps of 19 years between 1960 and 1985 where there were notable storms and wrecked terns (Fig. 5). The impact of hurricanes appears to have a large geographical range. Individuals were wrecked as far south and east as the Lesser Antilles to as far north as New England in the United States. Hurricanes can sweep birds for hundreds of kilometers along the storm’s track such as in the case of Hurricane Donna in 1960 along the east coast of the United States.

**Table 3:** Table of distribution of recovered wrecked birds. The number of wrecked birds are broken down by the region they were found in and listed with the notable hurricanes of the year.

Year	# Wrecks	N America	Caribbean	Central America	Notable Hurricanes
1960	9	6	2	1	Abby/Donna
1961	6	6	0	0	Carla
1962	0	0	0	0	
1963	2	0	2	0	Flora
1964	6	4	2	0	Cleo/Hilda
1965	1	1	0	0	Betsy
1966	1	1	0	0	Inez
1967	7	0	4	3	Beulah
1968	3	3	0	0	Abby
1969	7	7	0	0	Camille
1970	3	2	1	0	Becky^a^
1971	3	0	0	3	Edith
1972	2	1	1	0	Agnes
1973	12	8	1	3	Brenda/Delia^a^
1974	5	2	1	2	Carmen
1975	4	2	0	2	Caroline/Eloise
1976	0	0	0	0	
1977	0	0	0	0	
1978	0	0	0	0	
1979	10	6	4	0	Bob/David
1980	21	18	1	2	Allen
1981	0	0	0	0	
1982	2	2	0	0	Alberto
1983	0	0	0	0	
1984	0	0	0	0	
1985	2	2	0	0	Elena/Gloria

**Notes.**

a A tropical storm instead of a hurricane.

**Figure 6:**
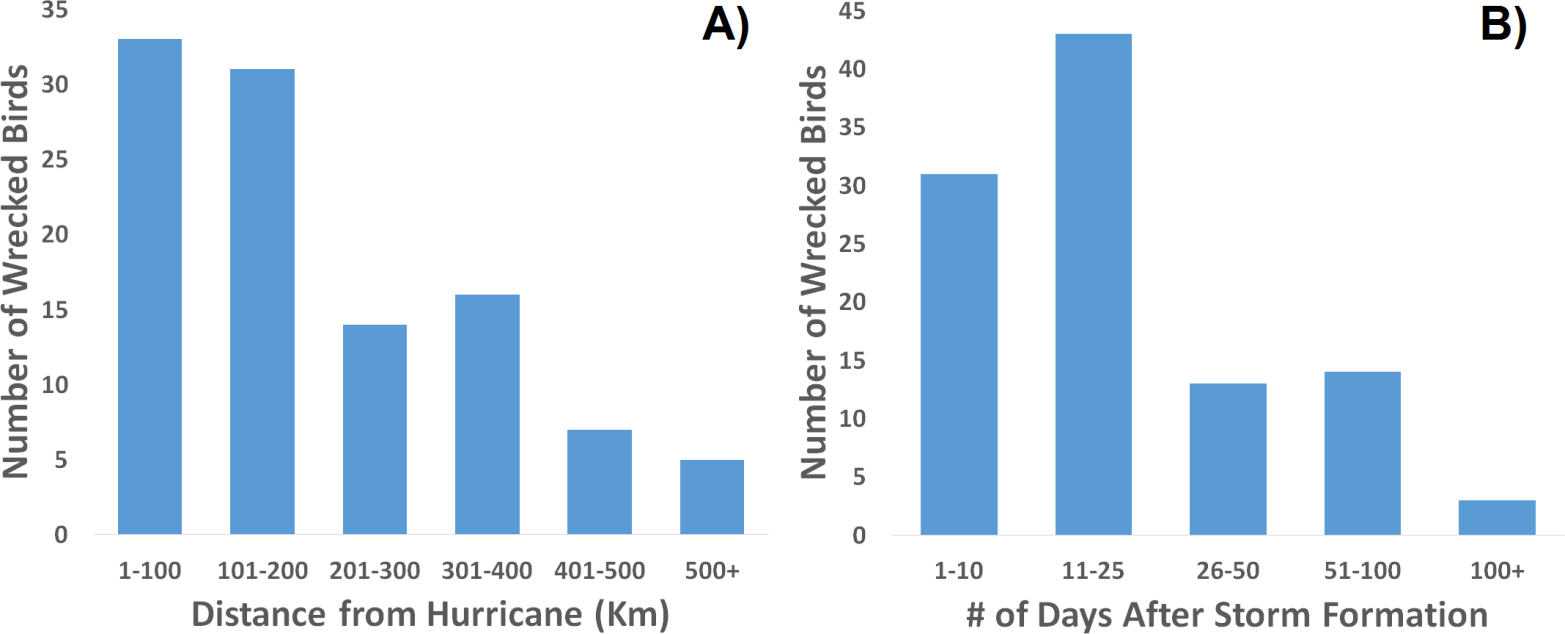
Frequency distribution of recovered, wrecked birds in relation to hurricanes of the season. Wrecks are more often recovered (A) closer to a storm’s center and (B) sooner after the storm’s formation.

**Figure 7:**
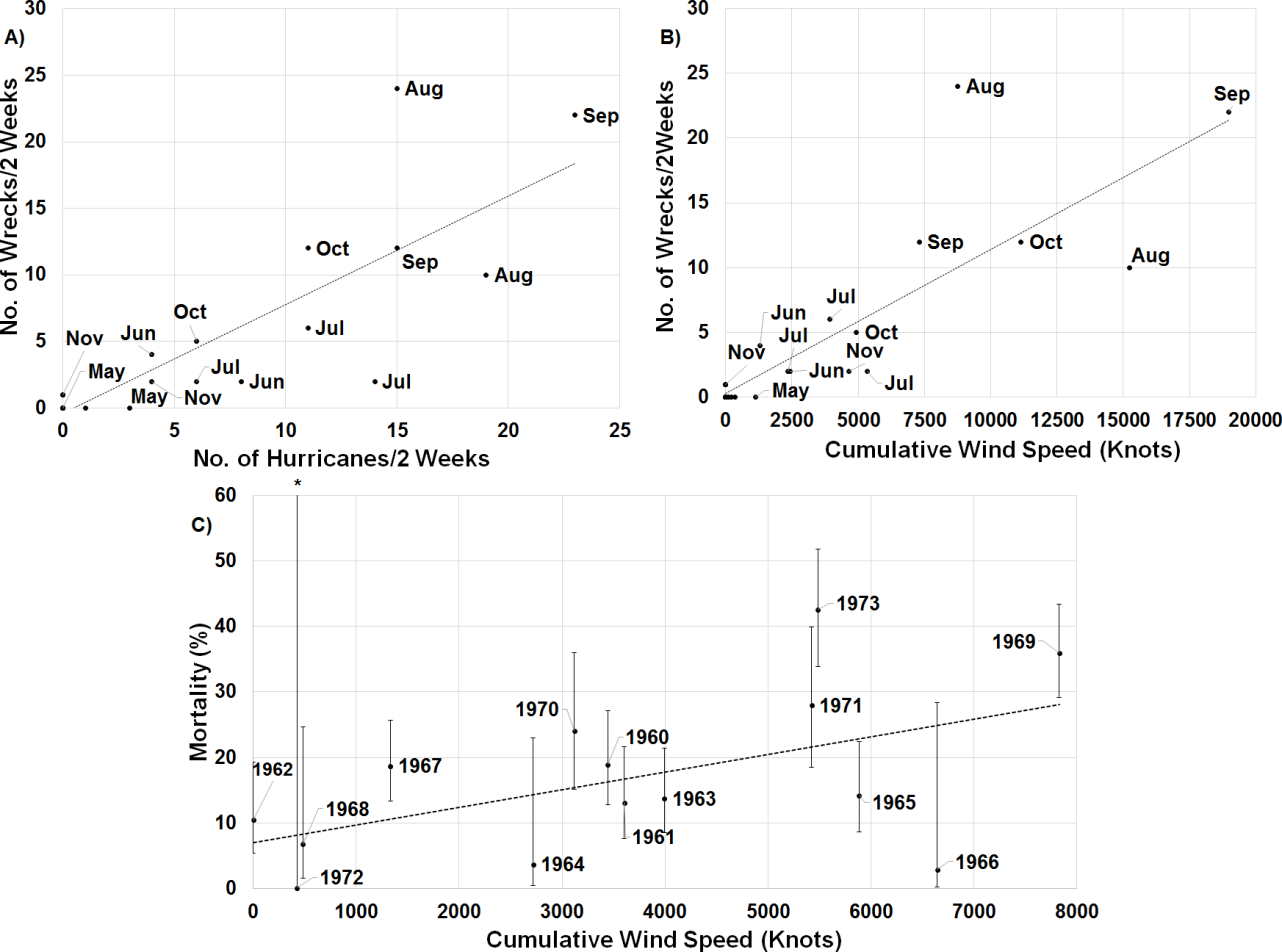
Linear regressions between estimates of mortality and hurricane frequency and intensity. There is a strong positive relationship between the number of wrecked individuals in a two-week period and (A) the total number of hurricanes category 3 and higher in the region of interest during that time (*y* = 0.816*x* − 0.381, *r*^2^ = 0.708, *p* < 0.0001; one-tailed) and (B) the cumulative wind speed of the same set of hurricanes (*y* = 0.001*x* + 0.307, *r*^2^ = 0.725, *p* < 0.0001; one-tailed). The points are broken down by month to illustrate the impact of hurricanes throughout the year. (C) Annual apparent mortality from the wind-constrained year only MARK model also shows a weak but significant (*y* = 0.002*x* + 6.997, *r*^2^ = 0.238, *p* = 0.033; one-tailed) relationship with cumulative wind speed of these hurricanes throughout the year. Also shown are the 95% confidence intervals of the mortality estimates provided by MARK. ^∗^ indicates the confidence interval stretches beyond the axis to a value of 100.

When one maps the locations of all wrecked individuals in a given year, what stands out is that all locations were associated with a tropical storm (Fig. 5). This is supported by the trend that there were more wrecks closer to the estimated wind radii and within a few weeks of a storm’s formation (Fig. 6). Furthermore, when we aggregate all category 3 and higher storms across the region and time of interest to avoid singling out storms, we see this trend continue. There is a strong positive correlation between the number of wrecked individuals in a two-week period and both the number of storms in that period (Fig. 7A, *r*^2^ = 0.708, *p* < 0.0001; one-tailed) and the cumulative wind speed (Fig. 7B, *r*^2^ = 0.725, *p* < 0.0001; one-tailed). This effect is predominant at the height of hurricane season, from August to October. When we replace the number of wrecked individuals with the mortality estimates derived from the MARK model, we still find a positive relationship between cumulative wind speed and mortality, albeit a weak but significant one (Fig. 7C, *r*^2^ = 0.238, *p* = 0.033; one-tailed). While we account for individual variance using a weighted regression, we do not adjust for covariance between years in this analysis. The greatest absolute value of correlation between years was 0.0019 which we consider small enough to dismiss.

## Discussion

Prior to this study, there was no published information on where sooty terns migrated to after the breeding season. With the use of tracking technology, we have been able to map some sooty tern migrations into the Atlantic Ocean. We recognize that migration patterns may vary across time and individuals (Quillfeldt, Voigt & Masello, 2010; McFarlane Tranquilla et al., 2014) and that a larger sample size and study period is necessary to determine consistency of migration routes. Nonetheless, we now know that some individuals travel from the Florida Keys, through the Caribbean Sea, along the coast of South America, and winter in the mid-Atlantic. In addition, the presence of deceased individuals along the same route suggests that many more do the same.

Another limitation of the sample size is that we were unable to temporally stagger deployment of transmitters throughout the season which may impact our understanding of the timing of individuals’ movements. We do not however, expect that this has impacted our understanding of the spatial extent of sooty tern movements given that our telemetry results match locations from recovered individuals.

Fisheries have complex impacts on seabird survival; from disrupting marine communities and trophic cascades (Garthe & Scherp, 2003; Wickliffe & Jodice, 2010), to providing easily accessible by-catch (Garthe, Camphuysen & Furness, 1996), to deaths caused by fishing equipment (Tasker et al., 2000; Furness, 2003). In particular, we thought the local fishing industry might impact sooty terns. The minimal overlap between shrimping locations and tern foraging locations suggest that this is not a regular occurrence. Not only is the area of overlap small, but it also disproportionately covers areas we have deemed unimportant for feeding among tagged individuals. This is in contrast to brown noddies (*Anous stolidus*), a similar species that cohabit the Dry Tortugas with sooty terns during the breeding season, whose foraging grounds overlap up to 93.3% with commercial fishing areas (Maxwell et al., 2016). Similar to our limitations in recording individual variation in migration patterns, we acknowledge that our sample size does not fully encapsulate the full extent of spatial and temporal variation of feeding grounds, but present these data as an important first step in documenting such movements.

We previously considered danger from future oil spills in the area as another potential threat via either direct mortality or from food web disruption (Moreno et al., 2013). At first one might ask what is the purpose of comparing seabird movement with an oil spill from three years prior. Without the benefit of foresight, our post-hoc approach represents a measure of the potential a spill like Deepwater Horizon might have had in the past, or might have in the future. While the furthest extent of the terns’ movements during the breeding season had a small amount of overlap with the furthest projections of spillage from the Deepwater Horizon event, surface oil from this particular event does not significantly overlap areas used by our tagged sooty terns. However, if such a spill from so far away could have reached areas used by this population, future spills that are closer or more catastrophic would likely also impact these areas. Again, sample size limitations mean that what we have presented here may not be the full picture and that our studied individuals only represent the minimum impact such an oil spill may have.

Although our average estimate of mortality (0.16) is higher than the 0.09 found by Feare & Doherty Jr (2004) in sooty terns in the Seychelles, it is within the range of mortalities generated by similar models for inshore species such as common terns (*Sterna hirundo,* 0.12–0.24, Breton et al. 2004, 0.065–0.209, Szostek & Becker, 2015), least terns (*Sternula antillarum*, 0.07–0.20, Renken & Smith, 1995), and roseate terns (*Sterna dougallii,* 0.09–0.26, Spendelow et al., 1995). Nonetheless, our highest estimate of mortality (0.41 in 1973) is higher than the highest reported tern mortality we found (0.34 for the sandwich tern, *Thalasseus sandvicensis*, Møller, 1983). Such a high mortality in a species that typically lays a single egg in a clutch (Feare, 1976) should lead to an overall decrease in population size. It is possible such as a decrease is occurring at the Dry Tortugas as counts of nesting pairs have decreased over recent years (Cope, 2016).

Previous studies have shown that tropical storms impact nesting and breeding success of sooty terns at the colony. White, Robertson Jr & Ricklefs (1976) found an increase in mortality and decrease in growth of chicks after Hurricane Agnes in 1972. The results of our study suggest hurricanes also kill and wreck birds along their migration path (Figs. 5 and 6). These direct mortality events appear to have a strong relationship with the number and intensity of storms (Fig. 7). Particularly strong hurricanes that cross through a large portion of the tern’s migratory route can have devastating impacts. The largest mortality estimated from recoveries of wrecked birds occurred in 1979 and 1980 (Fig. 4) when category 5 hurricanes David and Allen wrecked a large number of birds (Table 3). Hurricanes can also affect tern populations by carrying individuals to ill-favored locations. Hurricane Camille in 1969 swept an individual as far away as the Great Lakes between the US and Canada.

While the connections between particular storms, the fraction of wrecked birds, and the annual estimates of mortality are sometimes compelling, there are exceptions that we do not understand. First, our results imply that even weak tropical storms can lead to increased tern death. 1973 was overall a particularly calm year for hurricanes, but the number of wrecked individuals that year, as well as a spike in mortality (Fig. 4), indicates that tropical storm Delia may have killed many individuals. Evidence for the impact of this storm comes from the density of wrecked birds in a relatively small area, near where the storm came ashore in Texas.

Conversely, Hurricanes Cleo and Hilda wrecked birds, but 1964 was a year of low mortality. Cleo in late August decreased to a category 1 as it headed almost due north over central Cuba and then Florida, so perhaps the birds were still in the Gulf of Mexico or even off the coast of South America. Hilda came though the Gulf, but not until the end of September, when the birds might have been farther south and east. Similarly, 1966 was a year of low mortality but high cumulative wind speed (Fig. 7). Hurricane Inez is responsible for most of the accumulated wind speed, but did not form until late September, and thus potentially missed many individuals who were already further south. Nonetheless, the chances of terns being in the wrong place at the wrong time are expected to increase as cyclone frequency increases due to climate change (Bender et al., 2010).

In summary, we have presented the first ever movement data on sooty terns away from a colony, identifying areas used both during the breeding season as well as during the migratory route. These data suggest only a limited overlap with areas affected by anthropogenic threats. Natural disturbances in the form of tropical storms, however, appear to have a more significant relationship with adult tern mortality; the number of wrecked individuals is strongly correlated with both the number and intensity of storms in the same period. Overall mortality only shows a weak, but still positive relationship with storm intensity. This suggests that additional factors are at play. What seems likely is that it is not simply the strength of a hurricane, but that timing and location also play a role. By combining these aspects of storm occurrence with other considerations such as food availability and possible emigration rates, future studies may yield a more comprehensive understanding of adult sooty tern mortality.

##  Supplemental Information

10.7717/peerj.3287/supp-1 Supplemental Information 1 Supplemental Information

10.7717/peerj.3287/supp-2 Supplemental Information 2 Capture history of all individuals Juveniles have already been edited to have their first encounter removed. As such, all individuals have their adult capture history represented here

## Additional Information and Declarations

### Data Availability

The following information was supplied regarding data availability: Movebank Sooty terns (*Onychoprion fuscatus*) Dry Tortugas ID #: 16315427
https://www.movebank.org/panel_embedded_movebank_webapp.
