# Supplementary material for: Sooty tern (Onychoprion fuscatus) survival, oil spills, shrimp fisheries, and hurricanes
Source: PeerJ. 2017 May 10;5:e3287. doi: 10.7717/peerj.3287 (PMC5428334; doi:10.7717/peerj.3287)
Supplement: Supplemental Information 1 [file peerj-05-3287-s001.docx]

**Supplemental Information**

*Methods:*

Although there are commercial packages to estimate annual survivorship, we find they lack computational transparency. Moreover, by design they are general applications intended for all ranges of survival and detectability. For this study, we expected that survivorship would be relatively high — terns are long-lived birds — but the chance of recapturing marked birds in a given year in a colony of ~100,000 birds would be small. These facts impose constraints on how best to estimate the parameters.

We created a model for survivorship using banding data from three different sets of years (Fig. S1A). This requirement emerges because we must estimate two parameters: the yearly survival to year i from year i-1, *s*_i_, and the probability we will detect the birds marked in year i, *d*_i_. In Fig. S1A, circles represent the number of individuals caught in a given year while squares represent the number of individuals never seen again. We have included examples of actual data from our estimate of survivorship in 1960 as the number in the center of each shape. The first column illustrates the first time a bird is caught and banded (circle A).

This cohort is divided into two groups of survivors; one that is not detected in the subsequent year (circle B) and one group of individuals caught for a second year in a row (circle C). Circle C consists of birds that both survived and were detected in the following year. (The probability of this is *s*_i *_ *d*_i_ that a bird survives to the next year *s*_i_ and we detect it *d*_i_).

To determine the detection rate *d*_i_, we look at the groups of individuals that were seen again later (circles D and E), thus proving that they were alive in the second year and consider the fraction that was detected in this year $E/(D+E)$). This provides the independent estimate of *d*_i_ from which we can now estimate *s*_i_ by simple arithmetic:

$$s_{i} =\left( \frac{C}{A} \right)/\left( \frac{E}{D+E} \right)$$

As a check, we also estimated *s*_i_ and *d*_i_ using maximum likelihood estimators employing the observed numbers of banded and captured birds. For the large sample sizes we employ here, the estimates did not differ. Finally, to estimate standard errors, we ran a Monte Carlo simulation with 100 iterations using the estimate probabilities as a based, using a binomial distribution to generate the numbers of birds recaptured in each class and then calculating the distribution of estimates. We discarded estimates based on small sample sizes when these simulation estimates showed the confidence intervals to be too broad to be informative.

As Fig. S1A exemplifies, the fraction of birds re-captured in the year following marking is small, because the colony is so large. In contrast, the fraction of birds re-captured eventually is much larger because the birds are long-lived. The smallest number — and so the one with the proportionately greatest sampling error — is E, the numbers of birds caught in the year following marking *and* caught in all of the subsequent years.

This means that sampling error of this class will have the greatest impact of the estimates of survival and, in particular, any biases may lead to consistent errors. *A priori*, the most problematic would be for some birds to be more likely to caught than others. Indeed, that appears to be the case. Upon separating captures of those often many subsequent years, we found that birds caught the year after banding (group E in Fig. S1A) have on average, a 14% higher likelihood of being recaptured than individuals not caught the year immediately after banding (group D in Fig. S1A). This pattern is a consistent one over almost all the years of the study. This result has the effect of increasing our estimate of detection and subsequently suppressing our estimate of survival.

To adjust for this sampling bias, we created two separate Poisson distributions, one for each of the D (Fig. S1B) and E (Fig. S1C) groups, to estimate the number of times an individual is recaptured. In order to estimate the number of individuals alive at least two years post banding but were never seen again (the probability of zero occurrences in the Poisson distribution), we ran a chi-square goodness of fit test and minimized the chi-square statistic. We then used the new estimated total of birds in the D and E groups to create a new estimate of detection and survival. Due to variable sampling effort across time, we smoothed annual variation by using a moving window of five years (two years before and two years after the year of interest) to calculate the Poisson distributions. We did not calculate confidence intervals for this adjusted estimate because of the increasing uncertainty given the addition of multiple parameters.

***
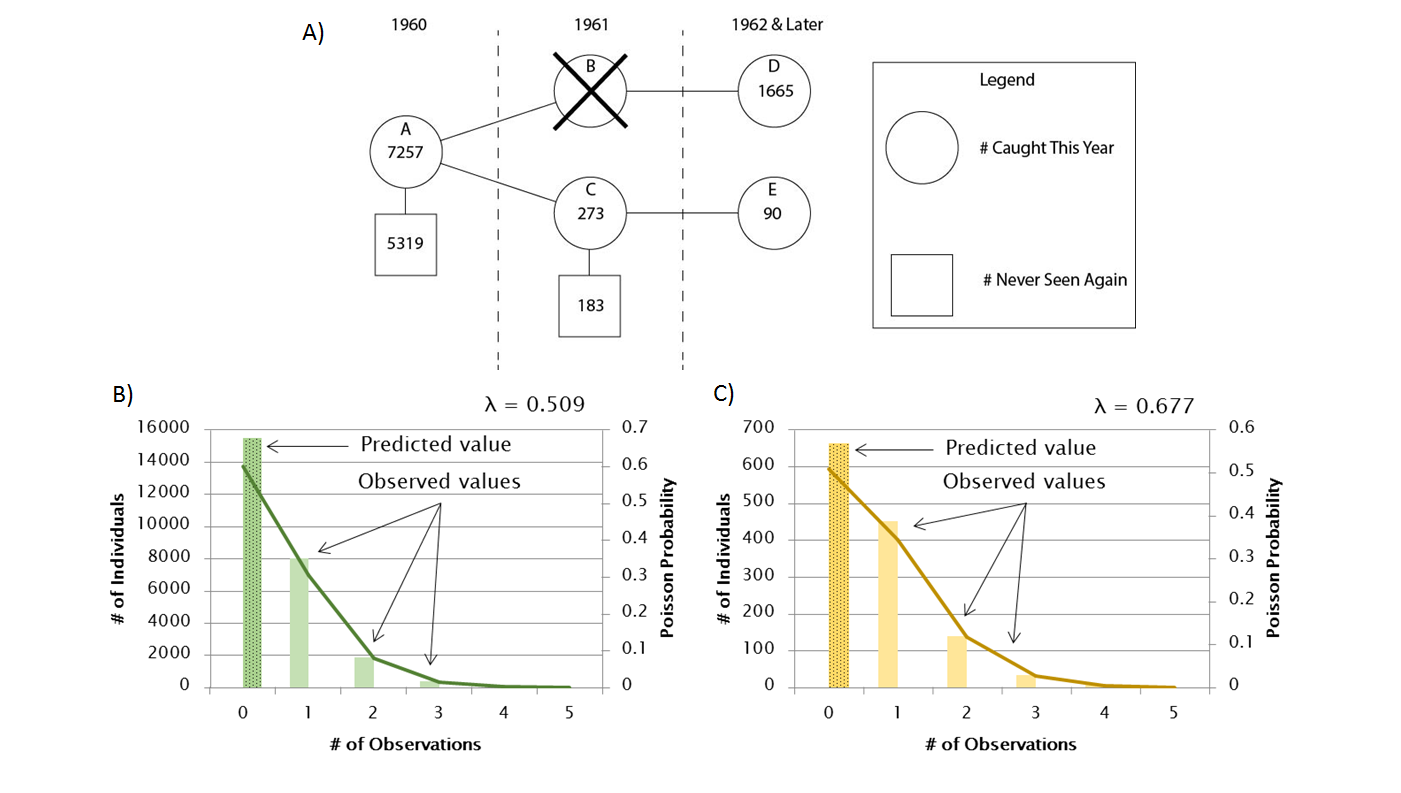
***

**Fig. S1:** **Visualization of the methods for calculating survivorship from banding data.** We first created a general model of survivorship based on banding data with an example from 1960 (A). To adjust for sampling bias, we fit a Poisson distribution to the number of observations of individuals caught once, twice, etc, two or more years after banding. We separated these individuals into those not caught the year after banding (B) and those caught the year after banding (C).

*Results:*

When using the model described above, we found that mortality varied from 10.35% in 1964 to 38.67% in 1973 (Fig S2). The average annual mortality and detection rate during this time were 25.01% and 6.10% respectively. 1967 had the highest number of individuals banded, but the highest detection rate occurred in 1966 (11.46%). Every year the terns potentially encountered a category 5 hurricane there is an associated spike in mortality. The highest mortality, however, occurred in 1973, when only tropical storm Delia intersected the likely path of the terns. Moreover, one of the three years with category 4 hurricanes (1964) showed the lowest annual mortality.

The adjusted estimates of mortality show a similar trend but are generally lower than the simulated estimates. As mentioned above, this is because we adjust for a sampling bias that increased estimates of detection and hence suppressed estimates of survival. Both of these estimates of mortality also match those reported by MARK but suggest an overall slightly higher rate of mortality.


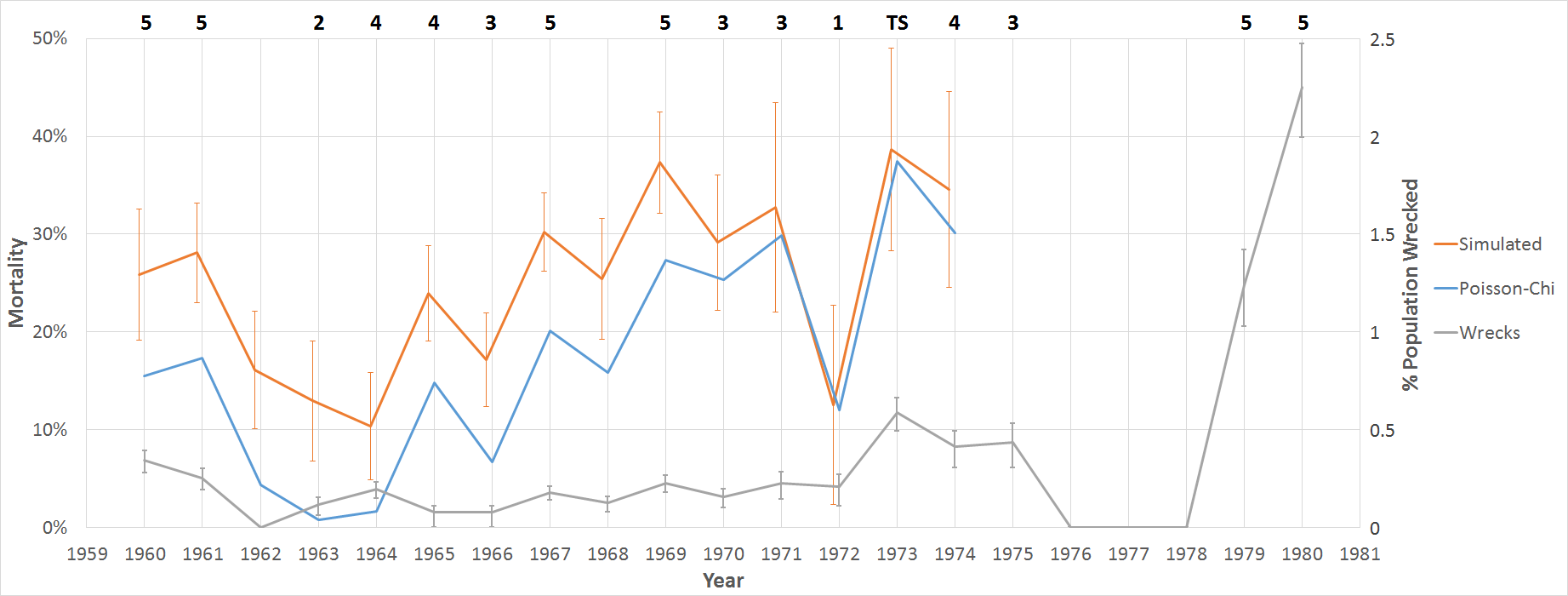


**Fig. S2:** **Graph of three estimates of tern mortality.** The orange line shows the original estimate from the survivorship model with standard errors generated by Monte Carlo simulations. The blue line is the adjusted estimate using a chi-squared minimized Poisson distribution. The gray line shows an independent estimate from recovered individuals. Across the top are the strongest classifications of notable cyclones for the year that intersected the sooty tern migratory pathway (TS denotes tropical storm).

**Table S1:** **Breakdown of recovered deceased sooty terns.** The following table is comprehensive breakdown of all 385 recovered individuals that were found in various regions for every year individuals were recovered.

| **Year** | **Africa** | **Central America** | **Caribbean** | **South America** | **USA** | **Year Total** |
| --- | --- | --- | --- | --- | --- | --- |
| 1940 | 0 | 0 | 0 | 0 | 1 | 1 |
| 1941 | 0 | 0 | 0 | 0 | 2 | 2 |
| 1942 | 0 | 0 | 1 | 0 | 0 | 1 |
| 1943 | 0 | 0 | 0 | 0 | 1 | 1 |
| 1945 | 0 | 0 | 0 | 0 | 1 | 1 |
| 1949 | 0 | 1 | 0 | 0 | 0 | 1 |
| 1950 | 0 | 0 | 0 | 0 | 1 | 1 |
| 1960 | 0 | 1 | 3 | 0 | 11 | 15 |
| 1961 | 5 | 2 | 0 | 0 | 9 | 16 |
| 1962 | 0 | 1 | 0 | 1 | 0 | 2 |
| 1963 | 1 | 2 | 2 | 0 | 1 | 6 |
| 1964 | 1 | 1 | 4 | 0 | 4 | 10 |
| 1965 | 9 | 3 | 0 | 0 | 3 | 15 |
| 1966 | 3 | 0 | 0 | 1 | 1 | 5 |
| 1967 | 5 | 3 | 5 | 0 | 2 | 15 |
| 1968 | 9 | 1 | 3 | 2 | 6 | 21 |
| 1969 | 1 | 0 | 0 | 0 | 8 | 9 |
| 1970 | 2 | 3 | 7 | 0 | 3 | 15 |
| 1971 | 0 | 4 | 1 | 1 | 0 | 6 |
| 1972 | 4 | 3 | 9 | 1 | 3 | 20 |
| 1973 | 2 | 9 | 2 | 0 | 12 | 25 |
| 1974 | 2 | 3 | 2 | 1 | 5 | 13 |
| 1975 | 2 | 2 | 1 | 1 | 3 | 9 |
| 1976 | 8 | 1 | 2 | 1 | 1 | 13 |
| 1977 | 4 | 2 | 2 | 1 | 3 | 12 |
| 1978 | 7 | 0 | 0 | 0 | 6 | 13 |
| 1979 | 0 | 3 | 5 | 0 | 8 | 16 |
| 1980 | 3 | 6 | 3 | 0 | 22 | 34 |
| 1981 | 0 | 0 | 3 | 0 | 12 | 15 |
| 1982 | 3 | 2 | 1 | 0 | 3 | 9 |
| 1983 | 0 | 0 | 8 | 0 | 0 | 8 |
| 1984 | 2 | 1 | 1 | 1 | 0 | 5 |
| 1985 | 0 | 0 | 0 | 0 | 3 | 3 |
| 1986 | 0 | 1 | 1 | 0 | 1 | 3 |
| 1987 | 0 | 1 | 2 | 0 | 4 | 7 |
| 1988 | 0 | 0 | 8 | 1 | 1 | 10 |
| 1989 | 0 | 0 | 1 | 0 | 1 | 2 |
| 1990 | 0 | 0 | 0 | 0 | 1 | 1 |
| 1992 | 0 | 0 | 2 | 0 | 0 | 2 |
| 1993 | 0 | 0 | 0 | 1 | 0 | 1 |
| 1994 | 0 | 0 | 1 | 0 | 1 | 2 |
| 1995 | 0 | 1 | 0 | 0 | 1 | 2 |
| 1996 | 0 | 0 | 0 | 0 | 1 | 1 |
| 1997 | 0 | 1 | 0 | 0 | 1 | 2 |
| 1998 | 0 | 0 | 0 | 0 | 6 | 6 |
| 1999 | 0 | 0 | 0 | 0 | 1 | 1 |
| 2000 | 0 | 0 | 0 | 0 | 1 | 1 |
| 2003 | 0 | 0 | 0 | 0 | 4 | 4 |
| 2005 | 0 | 0 | 0 | 0 | 2 | 2 |
| **Region Total** | 73 | 58 | 80 | 13 | 161 | 385 |
